# Locus coeruleus features are linked to vagus nerve stimulation response in drug-resistant epilepsy

**DOI:** 10.3389/fnins.2024.1296161

**Published:** 2024-02-13

**Authors:** Alexandre Berger, Elise Beckers, Vincent Joris, Gaëtan Duchêne, Venethia Danthine, Nicolas Delinte, Inci Cakiroglu, Siya Sherif, Enrique Ignacio Germany Morrison, Andres Torres Sánchez, Benoit Macq, Laurence Dricot, Gilles Vandewalle, Riëm El Tahry

**Affiliations:** ^1^Department of Clinical Neuroscience, Institute of Neuroscience, Catholic University of Louvain, Brussels, Belgium; ^2^Synergia Medical SA, Mont-Saint-Guibert, Belgium; ^3^Sleep and Chronobiology Laboratory, GIGA-Cyclotron Research Center-in vivo Imaging, University of Liège, Liège, Belgium; ^4^Faculty of Health, Medicine and Life Sciences, School for Mental Health and Neuroscience, Alzheimer’s Centre Limburg, Maastricht University, Maastricht, Netherlands; ^5^Department of Neurosurgery, Cliniques Universitaires Saint-Luc, Brussels, Belgium; ^6^GE Center MR Applications, General Electric Healthcare, Diegem, Belgium; ^7^Institute of Information and Communication Technologies, Electronics and Applied Mathematics, Catholic University of Louvain, Louvain-la-Neuve, Belgium; ^8^Innoviris, Brussels Institute for Research and Innovation, Brussels, Belgium; ^9^Department of Neurology, Center for Refractory Epilepsy, Cliniques Universitaires Saint-Luc, Brussels, Belgium

**Keywords:** locus coeruleus, epilepsy, biomarker, magnetic resonance imaging, vagus nerve stimulation

## Abstract

The locus coeruleus–norepinephrine system is thought to be involved in the clinical effects of vagus nerve stimulation. This system is known to prevent seizure development and induce long-term plastic changes, particularly with the release of norepinephrine in the hippocampus. However, the requisites to become responder to the therapy and the mechanisms of action are still under investigation. Using MRI, we assessed the structural and functional characteristics of the locus coeruleus and microstructural properties of locus coeruleus-hippocampus white matter tracts in patients with drug-resistant epilepsy responding or not to the therapy. Twenty-three drug-resistant epileptic patients with cervical vagus nerve stimulation were recruited for this pilot study, including 13 responders or partial responders and 10 non-responders. A dedicated structural MRI acquisition allowed *in vivo* localization of the locus coeruleus and computation of its contrast (an accepted marker of LC integrity). Locus coeruleus activity was estimated using functional MRI during an auditory oddball task. Finally, multi-shell diffusion MRI was used to estimate the structural properties of locus coeruleus-hippocampus tracts. These characteristics were compared between responders/partial responders and non-responders and their association with therapy duration was also explored. In patients with a better response to the therapy, trends toward a lower activity and a higher contrast were found in the left medial and right caudal portions of the locus coeruleus, respectively. An increased locus coeruleus contrast, bilaterally over its medial portions, correlated with duration of the treatment. Finally, a higher integrity of locus coeruleus-hippocampus connections was found in patients with a better response to the treatment. These new insights into the neurobiology of vagus nerve stimulation may provide novel markers of the response to the treatment and may reflect neuroplasticity effects occurring in the brain following the implantation.

## 1 Introduction

Approximately 30% of epileptic patients will develop a refractory form of epilepsy or drug-resistant epilepsy (DRE), meaning that they do not respond to at least two antiseizure drugs administered at correct dosages (Kwan et al., 2010). When patients are refractory, a presurgical evaluation is conducted in a dedicated epilepsy center, to better localize and potentially remove or disconnect surgically the epileptogenic zone from the normal brain. However, patients with an epileptogenic focus in the eloquent cortex, or with a non-localizable, multifocal, or generalized epilepsy cannot benefit from this procedure. In these cases, vagus nerve stimulation (VNS) is one of the options as an adjunctive treatment. Several studies have shown that the response rate may vary across patients from 45 to 65% (Toffa et al., 2020), with a mean response rate of 53.53% (Wang et al., 2019). A systematic review that included the results of 78 previous studies reported that 8% of patients become seizure-free (Englot et al., 2016). Finally, the mean response rate to the treatment is known to increase over time after the implantation (Englot et al., 2016), possibly reflecting neuroplasticity effects. However, the mechanisms of action of this therapy and the biological requisites to become responder (R, ≥ 50% reduction in seizure frequency) are still not completely known.

The vagus nerve projects to the nucleus tractus solitarius (NTS) in the dorsomedial medulla, which in turns sends projections to an array of areas, including brainstem areas toward the rostral ventromedial medulla, parabrachial nucleus, raphe nucleus, and toward ascending structures, including the amygdala, the cerebellum, the hypothalamus, and thalamus (Fornai et al., 2011). Critically, the NTS also projects to the locus coeruleus (LC), a pair of small 2.5 mm diameter-by-15 mm long brainstem nuclei, that constitutes the main source of norepinephrine in the brain (Aston-Jones et al., 1986; Keren et al., 2009). Previous studies demonstrated the involvement of the LC in the antiseizure effects of VNS (Naritoku et al., 1995; Krahl et al., 1998; Groves et al., 2005; Dorr and Debonnel, 2006; Cunningham et al., 2008; Manta et al., 2009; Raedt et al., 2011; Shen et al., 2012; Hulsey et al., 2017; Berger et al., 2021). Chemically induced lesion of the LC using 6-Hydroxy-dopamine hydrobromide and an acute LC inactivation using lidocaine hydrochloride in rats, increased the seizure severity under active VNS compared to rats with an intact LC (Krahl et al., 1998). Moreover, animal studies using implanted intracerebral electrodes, revealed direct activation of the LC following VNS administration (Groves et al., 2005; Dorr and Debonnel, 2006; Manta et al., 2009; Hulsey et al., 2017). In addition, the increased activity of the LC with VNS was intensity-dependent (Hulsey et al., 2017). Immunochemistry studies probing the presence of neural activity markers also revealed a modulation of the LC with VNS (Naritoku et al., 1995; Cunningham et al., 2008; Shen et al., 2012). Finally, a > 70% increase in the concentration of extracellular norepinephrine (NE) in the hippocampus following VNS administration prevented the development of pilocarpine-induced limbic seizure in rats (Raedt et al., 2011), emphasizing the critical involvement of this neurotransmitter (and the LC) in the antiseizure effects of VNS.

The long-term suppression of seizure may arise from a VNS-induced neurogenesis, due to the release of NE in cortical areas and the hippocampus (Roosevelt et al., 2006; Revesz et al., 2008). An immunochemistry study conducted in rats reported an increased progenitor (i.e., stem cells) proliferation in the hippocampus (and more specifically in the dentate gyrus) following VNS administration, reflecting a VNS-triggered hippocampal plasticity (Revesz et al., 2008). Increased concentration of NE in the prefrontal cortex, as well as an increased expression of brain-derived neurotrophic factors and fibroblast growth factors in the hippocampus and cerebral cortex were further reported acutely following VNS in rats (Follesa et al., 2007).

Although more limited, efforts have been made to investigate the modulation of the LC-NE system in humans. The resting pupil diameter, which is thought to be linked to the activity of the LC (Joshi et al., 2016)—although still debated (Megemont et al., 2022), was increased in a VNS ON condition compared to a VNS OFF condition (Desbeaumes Jodoin et al., 2015). Moreover, VNS elicited a peak pupil dilation with longer latency compared to a control condition that consisted in a somatosensory stimulation (Vespa et al., 2022). At the behavioral level, increased inhibitory performance, which is related to LC activity, was observed in a VNS ON condition compared to a VNS OFF condition (Schevernels et al., 2016). Using a behavioral test aiming at measuring the ability to suppress irrelevant information, van Bochove et al. (2018) found similar findings but in clinically determined R to VNS only. Likewise, at the electrophysiological level, the amplitude of the P3 evoked-related potential during an auditory oddball task, an EEG-measured brain response known to be associated with LC activity (Nieuwenhuis et al., 2005; Murphy et al., 2011), was increased in a VNS ON condition compared to a VNS OFF condition in R only (Brazdil et al., 2001; De Taeye et al., 2014; Hödl et al., 2020).

Despite all these neurobiological insights, there is a critical need to refine our current knowledge about the LC-dependent mechanisms of action of VNS and explore possible differences between R and non-responders (NR, < 30% seizure frequency reduction). The development of specific MRI sequences allowed to visualize the LC *in vivo* and extract its contrast—a LC feature that is known to reflect the integrity of the nucleus (Betts et al., 2019). The primary goals of this pilot study were to test whether magnetic resonance imaging (MRI)-based LC contrast and functional response were related to the therapeutic efficacy of VNS. We further explored whether these LC characteristics were related to the duration of VNS therapy in search of potential neuroplasticity effects. In addition, we assessed whether the structural integrity of the white matter tracts arising from the LC and projecting to the hippocampus was related to the therapeutic efficacy of VNS.

## 2 Materials and methods

### 2.1 Participants

Patients were recruited from the VNS database of the Center for Refractory Epilepsy of Saint-Luc University Hospital, Brussels, Belgium. The patients met the following criteria: (i) DRE diagnosis, (ii) treated with VNS (DemiPulse^®^ Model 103 or DemiPulse Duo^®^ Model 104, AspireHC^®^ Model 105 or AspireSR^®^ Model 106; LivaNova, Inc., London, UK) for at least six months, (iii) able to understand the study protocol and (iv) aged 18 years or older. Exclusion criteria consisted in severe side effects of VNS reported by the patients such as dyspnea, pain in the neck/ear region, or gastrointestinal complaints, history of alcohol or drug abuse, the presence of psychiatric illnesses, hearing problem reported by the patients, travel in a country with a different time zone over the last month and any MRI contraindication. The clinical response to VNS was determined by the neurologist at the last follow-up visit. Based on these criteria, a sample of 23 patients were recruited, including 10 NR, 5 partial responders (PR) and 8 R, presenting, respectively, < 30%, 30–50% (with seizure-suppressing effects reported with the magnet mode–when swiping the magnet in front of the generator) and > 50% reduction in seizure frequency (Table 1). In 4 NR, the VNS was off for several reasons: (i) two patients were explanted 4 months and 2.4 years before the experiment, (ii) in one patient the device was turned off completely for almost 2 years due to side effects and a lack of response, and (iii) in one patient the battery was empty and not replaced since no positive effect was observed. All patients signed the informed consent prior to any investigation. The study received approval by the Ethical Committee of Saint-Luc University Hospital (reference nr. 2021/18FEV/086).

**TABLE 1:** Demographic and clinical characteristics of the study population.

Characteristics	NR (*n* = 10)	R/PR (*n* = 13)	*p*-value
Age (years)	35.1 ± 15.19	38.92 ± 11.22	0.49
Sex	6 females 4 males	7 females 6 males	1.00
Therapy duration of VNS (months)	91.1 ± 52.15	72.54 ± 83.54	0.54
Epilepsy type	10 focal 0 generalized	11 focal 2 generalized	0.48
Epilepsy duration (years)	24 ± 11.32	28.31 ± 13.62	0.49
Number of ASMs (number of patients)	2 ASMs: 4 3 ASMs: 3 4 ASMs: 3	2 ASMs: 6 3 ASMs: 6 4 ASMs: 1	0.22
Benzodiazepine (daily) intake (number of patients)	2	1	0.18
VNS intensity (mA)^a^	1 mA: 0/6 1.125 mA: 0/6 1.25 mA: 0/6 1.50 mA: 1/6 1.75 mA: 3/6 2 mA: 2/6	1 mA: 2/13 1.125 mA: 1/13 1.25 mA: 2/13 1.50 mA: 5/13 1.75 mA: 1/13 2.00 mA: 2/13	0.03
VNS frequency (Hz)^a^	20 Hz: 4 25 Hz: 2 30 Hz: 0	20 Hz: 5 25 Hz: 2 30 Hz: 6	0.15
VNS pulse width (μs)^a^	250 μs: 5 500 μs: 1	250 μs: 11 500 μs: 2	1.00
Rapid duty cycle^a,b^	0	2	0.91

^a^Values reported after excluding 4 NR (see text).

^b^The duty cycle is defined as (ON time + 4 s)/(ON time + OFF time), and a rapid duty cycle is defined as an OFF time ± 1.1 min, while keeping the duty cycle ± 50% (Kayyali et al., 2020). NR, non-responder; R, responder; PR, partial responder; ASM, antiseizure medication.

### 2.2 Oddball paradigm

Knowing that the auditory oddball task recruits the LC (Murphy et al., 2014; Berger et al., 2023), this paradigm was chosen to extract the functional response of the LC in a VNS OFF condition in the current study. The task was administered during functional MRI acquisitions and consisted of rare target stimuli (1,000 Hz sinusoidal waves, 100 ms) appearing approximately 20% of the time and pseudorandomly interleaved in a stream of standard sounds (500 Hz sinusoidal waves, 100 ms). Two-hundred and twenty stimuli were administered in total, including 46 target tones (interstimulus interval: 2,500 ms). The experimental paradigm was designed using OpenSesame 3.3.8 (Mathôt et al., 2012). MRI-compatible headphones (NeuroNordicLab, Bergen, Norway) were used to deliver the stimuli. Participants were instructed to press on a button box (ResponseGrip, NeuroNordicLab, Bergen, Norway) with the right index finger as soon as possible at the appearance of target sounds. A test sequence composed of 35 stimuli including 8 target sounds was realized during an MRI acquisition, to ensure an optimal perception of the stimuli and comprehension of the instructions.

### 2.3 Imaging parameters

The MRI acquisitions were realized following the LivaNova guidelines for MRI (setting the output current to 0 mA and turning off sensing). Imaging data were acquired using the SIGNA™ Premier 3T MRI system (GE Healthcare, Milwaukee, WI, USA), with a 48-channel head coil. T1-anatomical images were acquired using a magnetization prepared—rapid gradient echo (MPRAGE) sequence: TR = 2,186 ms, TE = 2.95 ms, FA = 8°, TI = 900 ms, bandwidth = 244.14 Hz, matrix size = 256 × 256, 156 axial slices, imaging frequency = 127.77 Hz, voxel size = 1 × 1 × 1 mm^3^, acquisition time = 5:26 min.

A 3D-high-resolution Magnetization Transfer weighted Turbo-FLash (MT-TFL) sequence was used to visualize the LC: TR = 69 ms, TE = 5 ms, FA = 20°, bandwidth = 65.12 Hz, matrix size = 512 × 512, 28 axial slices, voxel size = 0.39 × 0.39 × 1.7 mm^3^, acquisition time = 11:40 min. For this axial acquisition, the LC slab was centered perpendicularly to the rhomboid fossa, i.e., the floor of the fourth ventricle.

Blood-oxygen level-dependent (BOLD) functional MRI data were acquired with a multiband echo-planar imaging (EPI) sequence: acceleration factor = 3, TR = 1,700 ms, TE = 30 ms, FA = 90°, bandwidth = 3,906.2 Hz, matrix size = 128 × 128, 75 axial slices, voxel size = 1.7 × 1.7 × 2 mm^3^, number of volumes acquired = 335, acquisition time = 9:41 min.

Diffusion MRI data were acquired with a pulsed gradient spin echo (PGSE) sequence: TR = 4,837 ms, TE = 80.5 ms and flip angle = 90°. A high-gradient multi-shell diffusion scheme was used and consisted of 64 gradients at *b* = 1,000 [s ⋅ mm^–2^], and 32 gradients at *b* = 2,000, 3,000, and 5,000 [s ⋅ mm^–2^], interleaved with 7 b0 images. The in-plane field-of-view was 220 × 220 mm^2^ and the data contained 68 axial slices with a 2-mm thickness (no inter-slice gap, 2-mm isotropic voxels). A multi-slice excitation scheme was used during the acquisition with a hyperband slice factor of 3 to reduce the acquisition time. The total acquisition time was 13:33 min.

Finally, a T2-weighted image was acquired to improve the patient-specific segmentation of the hippocampus. The T2-weighted image was acquired using a spin-echo (SE) sequence: TR = 2.5 ms, TE = 91 ms, FA = 90°, matrix size = 255 × 255, 141 sagittal slices, voxel size = 1 × 1 × 1 mm^3^, acquisition time = 2:01 min.

### 2.4 Data analysis

#### 2.4.1 LC contrast extraction

**FIGURE 1:**
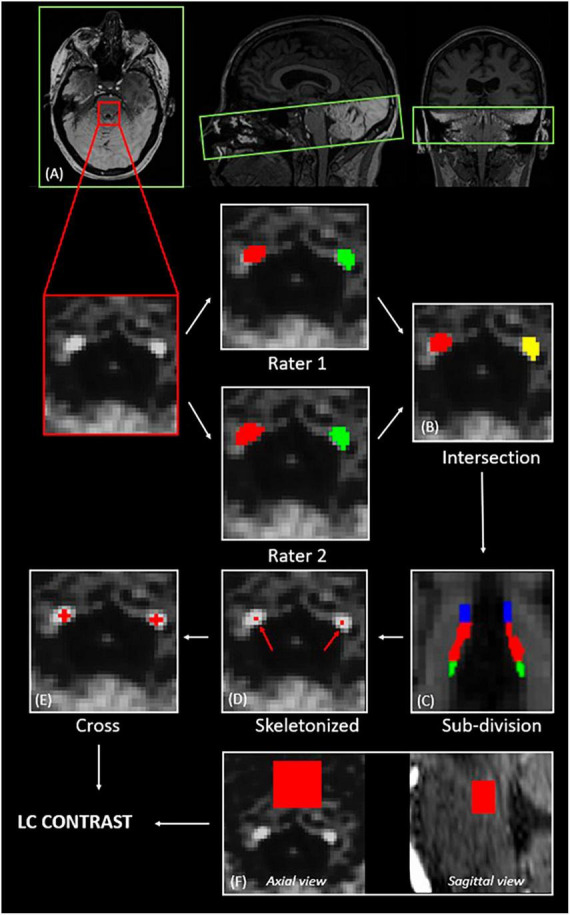
Extraction of the LC contrast. **(A)** Registration of the LC slab to the structural T1-anatomical image (axial, sagittal and coronal views); **(B)** computation of the intersection of the LC masks of two independent raters; **(C)** subdivision of the LC into rostral—25 upper % (blue), medial—50 middle % (red), and caudal—25 lower % (green); **(D)** selection of the voxel with the highest intensity for each axial slice; **(E)** centering of a 5-voxel cross on the voxel with the highest intensity; **(F)** definition of a slice-corresponding reference region in the pons (axial view), and final 3D reference region (sagittal view).

T1-anatomical images were upsampled by a factor 3, ending up with a resolution of 0.33 × 0.33 × 0.33 mm^3^, to avoid losing in-plane resolution when registering the LC slab to the T1 image. The MT-TFL image of each subject was registered to the whole brain upsampled T1 image by means of a two-step process: (i) a rough manual registration to extract the parameters for an initial transformation using ITK-SNAP (Yushkevich et al., 2006), and (ii) an automatic registration based on the initial transformation parameters, using the advanced normalization tools (ANTs, Penn Image Computing and Science Laboratory, UPenn, USA)^1^ (Figure 1A). Two trained raters (AB and EB) manually delineated the LC independently. The intersection of the LC masks of the two raters was computed (Figure 1B). Each LC mask was then divided into different subregions (25% rostral, 50% medial and 25% caudal portions of the LC) (Figure 1C). Other studies investigating LC integrity in different clinical conditions also used subdivisions of the LC, based on the fact that the LC is topographically organized in regions composed of cells projecting to different areas of the brain (Shibata et al., 2007; Dahl et al., 2019; Liu et al., 2020; Doppler et al., 2021; Calarco et al., 2022; Bell et al., 2023). It was suggested that the rostral part of the LC would project to the forebrain including the hippocampus, while the medial and caudal portions would project to the basal ganglia, cerebellum, and spinal cord (Pickel et al., 1974; Satoh et al., 1977; Mason and Fibiger, 1979; Loughlin et al., 1986). While no evidence currently exists on how VNS could differentially activate or modulate each of these subregions and how each region could be individually involved in the antiseizure effects of VNS, subdivisions of the LC were used in the present study to investigate more specific differences that could be hidden when considering the LC as a whole.

For each axial slice of the LC subpart masks, a 5-voxel cross was centered on the voxel with the highest intensity (Figures 1D, E). For each LC slice, the LC intensity was normalized to a slice-corresponding 2D reference region (a 15 × 15 voxels region, corresponding to a 4.95 × 4.95 mm^2^ square region) situated anteriorly to the LC in the pons, in the pontine tegmentum (Figure 1F). The complete reference region is therefore a 3D region composed of (number of LC slices × 15 × 15) voxels. The LC contrast was computed as the mean normalized intensities over the length of the LC.

Therefore, for each LC subpart, the contrast was computed as follows (e.g., for left LC):

$$C\,o\,n\,t\,r\,a\,s\,t\,L\,C_{L\,e\,f\,t-R\,o\,s\,t\,r\,a\,l}\,\frac{1}{n}\,\sum_{i=1}^{n=\left|L\,C_{L\,e\,f\,t-R\,o\,s\,t\,r\,a\,l}\right|}\left(\frac{{\bar{L\,C}}_{L\,e\,f\,t-R\,o\,s\,t\,r\,a\,l,i}}{{\bar{p\,o\,n\,s}}_{i}}-1\right)$$

where:

– *i* is the slice index along the LC subpart
– *n* is the number of slices along the LC subpart
– ${\bar{L\,C}}_{L\,e\,f\,t-R\,o\,s\,t\,r\,a\,l,i}$ is the mean intensity within the 5-voxel cross for the LC slice with index i
– ${\bar{p\,o\,n\,s}}_{i}$ is the mean intensity within the corresponding 2D reference region (15 × 15 voxels) for the LC slice with index i.

#### 2.4.2 Functional preprocessing and LC activity extraction

BrainVoyager (Brain Innovation B.V., Maastricht, Netherlands) was used to process EPI images. First, slice scan time correction was applied using the cubic spline interpolation. Three-dimensional motion correction was applied with a trilinear/sinc interpolation, using the first volume as the reference volume. Temporal high pass filtering was applied using the generalized linear model (GLM) approach with the Fourier basis set to remove slow-frequency drift. A default cut-off of 3 cycles was used, meaning that sine and cosine functions with cycles < 3 are modeled in the design matrix and removed from the voxel’s time course. Registration of functional MRI data to the structural space was realized after conducting brain extraction on the T1 image. For the registration, the initial alignment was conducted before a fine-tuning alignment based on the intensity of the images using multi-scale rigid-body transformations. The timing vector containing the appearance of target sounds was convolved with the standard two-gamma hemodynamic response function (HRF) and used as the main condition in a GLM. Realignment parameters (3 translations and 3 rotations) were used as multiple regressors of no interest. All predictors were z-transformed before fitting the model. A region-of-interest (ROI) analysis was conducted in the space of the subject. Based on the ROI signal time course, the beta values associated with the target appearance were extracted in the left and right LC subparts for each subject.

#### 2.4.3 Microstructural features of LC-hippocampus connections

Diffusion data of 18 patients were available, including data of 7 NR and 11 R/PR. Due to an amygdalohippocampectomy performed in the right hemisphere of one patient (a NR), LC-hippocampus connections were extracted in 17 patients for the right hemisphere, and in 18 patients for the left hemisphere. Data preprocessing was done using ElikoPy.^2^ Preprocessing steps included: skull stripping [using the Diffusion Imaging In Python library—DiPy,^3^ (Garyfallidis et al., 2014)], Rician denoising [using the Marcheko–Pastur Principal Component Analysis–MPPCA (Veraart et al., 2016)], Eddy currents, susceptibility distortion and motion corrections [Eddy command of the FMRIB Software Library—FSL^4^ (Andersson and Sotiropoulos, 2016)].

Diffusion tensor imaging (DTI) maps (fractional anisotropy: FA, mean diffusivity: MD, axial diffusivity: AD and radial diffusivity: RD) were reconstructed using DiPy. A multi-compartment fingerprinting model (also called Microstructure Fingerprinting - MF) based on Monte Carlo simulations of diffusion MRI signals was also used to extract quantitative microstructural features of white matter (Rensonnet et al., 2019). MF is a powerful multi-compartment model that does not make assumptions about the tissues and the diffusion processes (Rensonnet et al., 2019). MF estimates the fraction of occupancy of the crossing fascicles within each voxel, and their corresponding fiber volume fraction [or axonal density (Delinte et al., 2021)]. Therefore, the weighted fiber volume fraction (wFVF) value estimated in each voxel is defined as:

$$w\,F\,V\,F_{i}\,\frac{\nu_{1,i}*f\,v\,f_{1,i}+\nu_{2,i}*f\,v\,f_{2,i}}{\nu_{1,i}+\nu_{2,i}}$$

where:

– i is the index of the voxel
– ν_1,*i*_ is the fraction of occupancy of fascicle 1 in the voxel i (and ν_2,*i*_ for fascicle 2)
– *fvf*_1,*i*_ is the fiber volume fraction of fascicle 1 in the voxel i (and *fvf*_2,*i*_ for fascicle 2).

All diffusion maps were registered to the T1-structural space of the subject, after registering the FA map to the T1-weighted image using the *antsRegistration* function from ANTs and applying the transformation parameters to the other diffusion maps. In order to extract the mean diffusion metrics along the LC-hippocampus connections, DSIstudio^5^ was used for the reconstruction of the fiber direction, using generalized Q-sampling imaging (QSI) (Yeh et al., 2010) with a default diffusion sampling length ratio of 1.25. The left and right LC masks were dilated by one voxel in every direction using DSIstudio. Segmentation of the left and right hippocampi was conducted with Freesurfer (Linux—centOS version 7.2) in the structural space of the subject using the T1- and T2-weighted images to improve segmentation of pial surfaces. The dilated left and right LC masks were used as seed regions, with, respectively, the left and right hippocampal masks that were used as end regions. The quantitative anisotropy was used as the tracking index, and default lengths of minimum 30 mm and maximum 300 mm were used for the tractography. The deterministic fiber tracking was done with the Runge–Kutta method, using a seed count of 10 million as the terminative criterion. For each patient, the final tractography was visually and carefully inspected by a neurosurgeon (VJ), and false fibers were excluded to clean the tracts. The mean diffusion metrics (DTI: FA, MD, RD, AD and MF: wFVF) were then extracted in the left and right LC-hippocampus connections.

#### 2.4.4 Statistical analysis

Statistical analyses were conducted using RStudio (version 4.2.1). The demographic and clinical characteristics were compared between NR and R/PR using Mann–Whitney U-tests or the Fisher’s exact tests for categorical variables (Table 1). After conducting Shapiro-Wilk normality tests without rejection of the null hypothesis (normality of the data) for the LC contrast, LC activity and diffusion metrics in LC-hippocampus connections, two linear models (LM) were used to model these features in terms of response and therapy duration (total duration between the implantation day and the MRI acquisition), controlling for age, sex, antiseizure medication (ASM) intake, epilepsy duration and benzodiazepine intake [they may lead to cognitive dysfunctions ascribed to sedation or inattention (Stewart, 2005), making this covariate particularly important for an attentional task]. In this pilot study, two models were used to assess independently the relationship between the LC features and VNS response or therapy duration, to avoid possible multicollinearity issues. Indeed, it has been reported that response rate to VNS increases with therapy duration (Xu et al., 2022). In addition, while no difference in therapy duration was found between R/PR and NR in the present pilot study, the statistical approach considering independently VNS response and therapy duration in the models may be useful in studies with larger cohort of patients, where these two variables may not be independent.

False discovery rate (FDR) correction was conducted for each LC metric to correct for multiple comparisons. Results of the statistical tests were considered as significant for *p*_FDR_ < 0.05, while only a trend is considered for uncorrected *p* < 0.05. In order to avoid fitting problems with the LM due to possible multicollinearity of the predictors, the variance inflation factor (VIF) was computed for the predictors included in each model and VIF values were lower than 5 (low correlation between the predictors).

## 3 Results

### 3.1 Accuracy of the oddball task

Functional MRI data was not available for one NR, for whom MRI acquisitions were stopped after the structural acquisitions, due to the occurrence of a seizure during the acquisitions. The mean accuracy to the oddball task was 85.44 ± 29.94% when all patients were considered, which we consider reasonable given the patients population. Four patients (2 R and 2 NR) had relatively low accuracy to the task (< 90%). Excluding these patients led to an accuracy of 98.30 ± 2.58%. Results of the statistical analyses are reported for the complete sample of patients and with the reduced sample, including patients with a good accuracy to the task only (> 90% detection of target sounds). Group-level analysis of the general oddball effects for the validation of the paradigm can be found in Supplementary Materials 1, 2.

### 3.2 Locus coeruleus response

**FIGURE 2:**
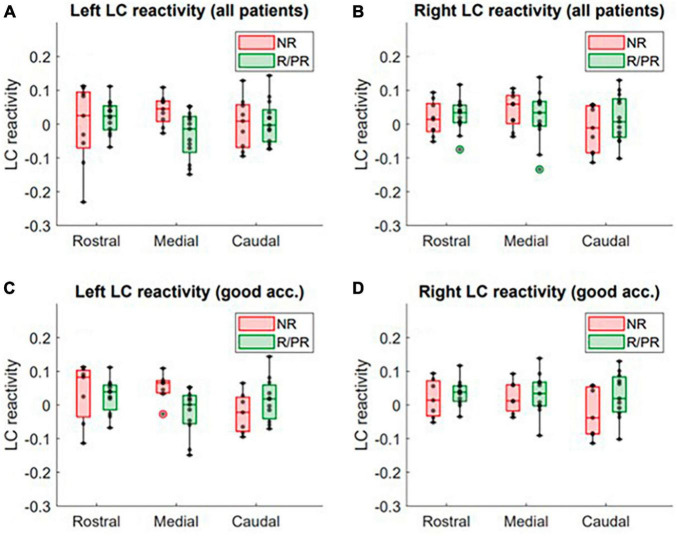
LC response to target stimuli in patients responding (or partially responding) and not responding to VNS treatment. Activity in the rostral, medial and caudal portions of the **(A)** left LC (all patients included), **(B)** right LC (all patients included), **(C)** left LC (only patients with a good accuracy to the task, i.e., > 90%) and **(D)** right LC (only patients with a good accuracy to the task, i.e., > 90%), in non-responders (NR) and responders (R)/partial responders (PR). Refer to text and Supplementary Material 3 for full statistical outputs of LM models.

The output of all the statistical models can be found in Supplementary Material 3. The LM using LC response as dependent variable, revealed a trend toward a significant relationship (not significant after FDR correction) between VNS response and LC response in the medial portion of the left LC (*N* = 22, *p* = 0.01*, *p*_FDR_ = 0.05 and *N* = 18, *p* = 0.03*, *p*_FDR_ = 0.11, when only patients with good accuracy are included), after controlling for age, sex, ASM intake, benzodiazepine intake and epilepsy duration (Supplementary Material 3A). For visual purposes, boxplots of the response in LC subparts in R/PR and NR are shown in Figure 2. No significant main effect of therapy duration was observed for the response in the medial portion of the left LC (*N* = 22, *p* = 0.72; and *N* = 18, *p* = 0.57, when only including patients with good accuracy) (Supplementary Material 3B).

### 3.3 Locus coeruleus contrast

**FIGURE 3:**
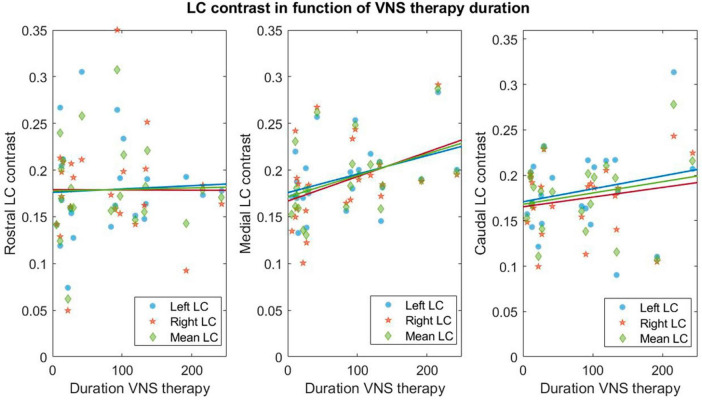
Linear regressions between contrasts in LC subparts and therapy duration. Linear regressions for the rostral–medial and caudal left (blue circles), right (orange stars) and mean over left and right (green diamond) LC and therapy duration (expressed in months). Refer to Supplementary Material 3 for statistical output of the LM.

A trend toward a significant relationship (not significant after FDR correction) was found between VNS response and LC contrast in the caudal portion of the right LC (*p* = 0.03*, *p*_FDR_ = 0.08) (Supplementary Material 3C). Moreover, a trend toward a significant effect of therapy duration on the contrast in the medial portion of the left and right LC was observed (left: *p* = 0.03*, *p*_FDR_ = 0.11 and right: *p* = 0.04*, *p*_FDR_ = 0.11—Supplementary Material 3D). For visual purposes, regressions between contrast of LC subparts and therapy duration are shown in Figure 3.

### 3.4 Locus coeruleus—hippocampus microstructure

**FIGURE 4:**
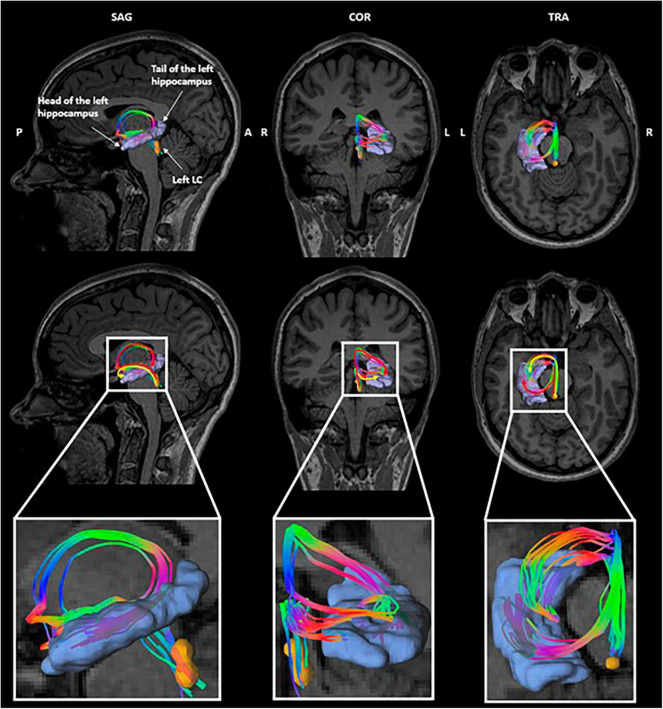
Tractography of the left LC-hippocampus connections in a representative subject. Orange: left locus coeruleus, purple: left hippocampus. The color of the tracts reflects the directionality of the fibers.

Due to the small size of the LC and the resolution of the diffusion data, the left and right LC masks were used without rostro-caudal subdivisions for the tractography. The tractography between the LC and the hippocampus yielded two bundles reaching, respectively, the head and the tail of the hippocampus (Figure 4). This corresponds to the described anatomy of the ascending noradrenergic bundles (Marien et al., 2004). Both bundles were considered jointly in our analyses. Individual tracking of LC-hippocampus connections realized in all patients can be found in Supplementary Material 4.

**FIGURE 5:**
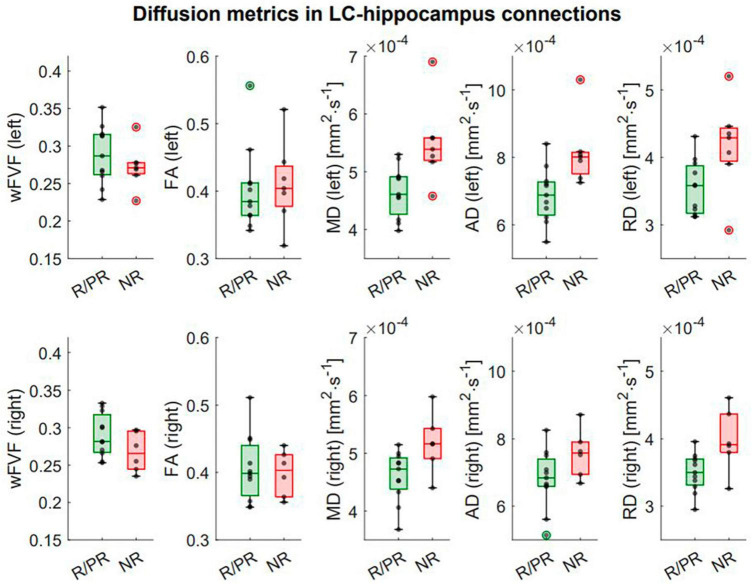
Integrity of LC-hippocampus connections in patients responding (or partially responding) and not responding to VNS treatment. First row: Boxplots showing the difference in diffusion metrics in left LC-hippocampus connections between responders (R)/partial responders (PR) and non-responders (NR). Second row: Boxplots showing the difference in diffusion metrics in right LC-hippocampus connections between R/PR and NR. Refer to text and Supplementary Material 3 for full statistical outputs of LM models.

In the left LC-hippocampus connections, a significant higher MD (*p* = 1.23e-3*, *p*_FDR_ = 3.07e-3*) and a higher AD (*p* = 0.02*, *p*_FDR_ = 0.03*) were found in NR (Figure 5). Moreover, a significant higher RD (*p* = 1.49e-5*, *p*_FDR_ = 7.43e-5*) and a lower wFVF (*p* = 0.02*, *p*_FDR_ = 0.03*) were found in the left LC-hippocampus connections of NR (Supplementary Material 3E). Finally, in the right LC-hippocampus connections, a significant higher MD (*p* = 0.01*, *p*_FDR_ = 0.03*) and higher RD (*p* = 0.003*, *p*_FDR_ = 0.01*) were found in NR. For visual purposes, boxplots showing the diffusion metrics in NR and R/PR are shown in Figure 5. No significant main effect of therapy duration on diffusion metrics was found (Supplementary Material 3F).

## 4 Discussion

Several animal and human studies suggested that the LC-NE system is a critical player in the antiseizure effects of VNS in patients with DRE (Roosevelt et al., 2006; Raedt et al., 2011; De Taeye et al., 2014; Hödl et al., 2020). Previous studies investigated non-invasive biomarkers of the modulation of the LC-NE system to demonstrate its implication in the antiseizure effects of VNS but also to understand the variability in response to the treatment across patients. However, no *in vivo* imaging study was conducted to directly assess the functional and structural characteristics of the LC and investigate potential differences in patients with DRE implanted with a VNS device.

The results of this pilot study suggested a trend toward a lower response in the medial portion of the left LC at the appearance of target sounds during an auditory oddball task in R/PR compared to NR to VNS (with the VNS being turned off). These functional findings may be in line with previous indirect assessment of LC-related measures. A previous study that measured the P3 event-related potential (i.e., a positive EEG deflection occurring around 300 ms post-stimulus) during an auditory oddball task found a higher amplitude in NR to VNS, in a VNS OFF condition (De Taeye et al., 2014). Another study that recorded the amplitude of the P3b component 2–4 weeks post-implantation but before any stimulation, revealed a lower baseline amplitude in patients who became R to VNS after 1 year of treatment (Hödl et al., 2020). Therefore, it could be hypothesized that patients with DRE show a different functional state of the vagal afferent network (and more specifically the LC-NE system), as suggested by Hödl et al. (2020). Individuals with a lower baseline activity could therefore constitute good candidates for a therapy targeting the LC-NE system, compared to individuals with a less modulable network.

Previous studies suggested rostro-caudal and antero-posterior regional specializations of the LC in terms of cell types and projections (Poe et al., 2020). Whether the link between response to VNS therapy and LC activity found in the medial portion of the left LC reflects a true lateralization or is due to statistical insensitivity over the right LC is unclear. However, knowing that the left vagus nerve (i.e., the VNS implantation side) projects–mainly, but not only–to the ipsilateral NTS, one could expect an effect mostly localized in the left LC. The NTS projects to the LC through two disynaptic pathways: an excitatory pathway with a relay within the nucleus paragigantocellularis (NPGi) (Reyes and van Bockstaele, 2006) and an inhibitory pathway with a relay within the nucleus prepositus hypoglossi (NPH) (Ennis and Aston-Jones, 1988, 1989; Luppi et al., 1995). These anatomical connections were confirmed through a stimulation study of the LC, where antidromic activations of the NPGi and NPH were observed (Aston-Jones et al., 1991). Since no known monosynaptic connections have been reported between the left and right LC (Luppi et al., 1995), it has been suggested that the contralateral response recorded when the LC is stimulated unilaterally, may be due to a putative interplay between the excitatory and/or inhibitory drive generated from the LC stimulation, via an antidromic activation of bilateral brainstem afferents (Marzo et al., 2014). Tracing studies revealed bilateral interconnections between the two NPH (Mccrea and Baker, 1985) and the two NPGi (van Bockstaele et al., 1989).

Therefore, it is not surprising that VNS may modulate the brain bilaterally, as potentially reflected in the trends toward an effect of therapy duration on the contrast in the medial portion of the left and right LC. Studies conducted in rats suggested that the NPGi projects to the medial (and ventral) portion of the LC (Schwarz and Luo, 2015). Moreover, studies found that thalamic-projecting cells were mainly located caudally in the LC of rats (Schwarz and Luo, 2015). Interestingly, we found a trend toward a relationship between LC contrast in the caudal portion of the right LC and VNS response. Whether the trend observed in the caudal right portion is a true regionalization or is due to noise-related sensitivity issues in the other portions, remains to be explored. Although translation of animal results to humans is not straightforward, our results together could possibly suggest that the functional and structural characteristics of the medial and caudal portions of the LC could differ between patients with DRE and may reflect modulatory effects of this therapy on the LC-NE system.

The exact biological interpretation of LC contrast as observed using specific MRI sequences remains debated (Galgani et al., 2021). Previous studies suggested that a higher LC contrast as observed using specific T1-weighted MRI sequences, was associated with a higher concentration of neuromelanin (a by-product of NE that accumulates inside the cell body of noradrenergic neurons), as confirmed in a study that used a combination of histology and post-mortem MRI (Keren et al., 2009). However, a recent study showed that LC contrast was still observable on images obtained using a sequence with magnetization transfer in mice that were genetically engineered to have 70% fewer LC cells (Watanabe et al., 2019). It has been suggested that MRI sequences with magnetization transfer effects (such as the sequence used in the present study) may set the LC apart from its surroundings due to the specific transfer of magnetization between different pools contained in noradrenergic neurons, i.e., the intracellular water content of the LC with the presence of paramagnetic ions, such as neuromelanin (Betts et al., 2019; Watanabe et al., 2019). Overall, and despite the current debate and investigations, the LC contrast is accepted as an indicator of LC integrity (Betts et al., 2019), with a higher contrast reflecting a better integrity depending on the age of assessment and excluding potential prodromal stages of degenerative brain disorders (Hansen, 2021; Jacobs et al., 2021). Therefore, the biological interpretation of our finding showing a trend toward an increased contrast in the medial portion of the LC over time with VNS may be that VNS triggers plastic effects that could improve or restore LC functions. Future studies, including longitudinal assessments, are needed to elucidate this question.

Bilaterally, we found a significant lower MD and RD in the LC-hippocampus connections in R/PR compared to NR. Moreover, a lower AD and higher wFVF were found in R/PR compared to NR, mainly localized in the left LC-hippocampus connections. These diffusion-related findings suggested a higher integrity of the LC-hippocampus connections in patients with DRE with a better response to the treatment. Indeed, it has been suggested that a lower MD and RD indicated healthier axons (Beck et al., 2021; Porat et al., 2022), and a higher fiber volume fraction reflected a higher axonal density (Delinte et al., 2021). While DTI metrics showed higher statistical differences between R/PR and NR than the MF metric, wFVF is known to be biologically interpretable and specific to the microstructural features of the fibers. Indeed, this model provides near ground truth for diffusion-weighted MRI signals for crossing fascicles (Rensonnet, 2019), while DTI is not able to capture characteristics of crossing fibers individually and is based on a simplified model of diffusion (a larger diffusion tensor is estimated for crossing fascicles). Therefore, MF offers supplementary information by disentangling the individual influence of crossing fascicles and confirming the interpretation of DTI metrics.

A previous tracing study that used horseradish peroxidase suggested the existence of strong fiber connections between the LC and the hippocampus in rats (Loughlin et al., 1986; Hansen, 2017). Another study conducted in non-human primates suggested that connections between the LC and the hippocampus could exist through an indirect pathway that includes the central tegmental tract and the medial forebrain bundle projecting toward the amygdala (Bowden et al., 1978). Structural and functional inter-connections between the amygdala and the hippocampus have been extensively reported in previous studies conducted in primates and rats (Saunders et al., 1988; Canteras and Swanson, 1992; McDonald, 1998; Pitakänen et al., 2000; Meisner et al., 2022), making the amygdala a potential relay between the LC and the hippocampus in the vagal afferent network. We used default tracking parameters (e.g., the step size or the angle threshold) which are known to limit the occurrence of false-positive tracts. In addition, the tracts were visually inspected to avoid tracts with unrealistic shapes. The connections highlighted in the present study were visually consistent across patients, providing further support that they do not reflect spurious tracts. However, quantifying the coherence of the tracts at the group level poses a challenge due to the inclusion of patients with congenital brain malformations, or patients who underwent brain surgery. This complexity precludes the use of a common space and constitutes a limitation in the present study. Future studies should nevertheless further investigate the consistency between tractography and postmortem data in humans.

In previous studies, an intensity-dependent increase of NE was measured in the hippocampus of healthy rats following VNS administration (Roosevelt et al., 2006), and a sufficient increase–of at least 70%, prevented the development of pilocarpine-induced seizures in rats (Raedt et al., 2011). Furthermore, NE concentration has been associated with the proliferation of neural progenitors in the hippocampus (Malberg et al., 2000; Kulkarni et al., 2002). Therefore, one could speculate that a better integrity of the tracts arising from the LC and projecting to the hippocampus, may allow a higher release of NE within the hippocampus during VNS administration and could be associated with a better response to the treatment and/or to greater neuroplasticity effects onto hippocampal structures.

Although our sample is acceptable for a population of patients with DRE and implanted with a VNS device, it remains relatively small for statistical analyses. In addition, including a sample of healthy subjects could be interesting to compare structural and functional characteristics of the LC with VNS-implanted DRE patients to gain further insight into the specific characteristics of this system in DRE and better understand the biological requisites to become R to the therapy. On a methodological level, respiratory and heart rate signals could be recorded in future studies, to regress out possible cofounding effects of physiological noises for the functional MRI analyses. When exploring the LC contrast, analyses could include a standard reference region for the normalization to minimize possible errors linked to the manual placement of the region and increase reproducibility of the procedure (Langley et al., 2017; Wengler et al., 2020; van der Pluijm et al., 2021).

Future longitudinal studies are needed to assess the evolution of the LC contrast/activity and the structural integrity of the LC-hippocampus connections over time after the implantation of a VNS device and compare the pre- and post-implantation characteristics. Such studies would provide deeper insight into the modulatory effects of the therapy on the LC and could help to establish new biomarkers of VNS efficacy. Other mechanisms of action for the seizure-suppressing effects of VNS have been previously suggested. Exploring the structural and functional characteristics of the dorsal raphe nucleus (a nucleus composed of serotoninergic neurons), or the concentration of inhibitory (e.g., gamma-aminobutyric acid) and excitatory (e.g., glutamate or aspartate) metabolites in the brain of DRE patients using spectroscopy, could serve as a promising direction for future investigations (Hammond et al., 1992; Krahl and Clark, 2012; Sarlo and Holton, 2021).

## Data availability statement

The raw data supporting the conclusions of this article will be made available by the authors, without undue reservation.

## Ethics statement

The studies involving humans were approved by the Ethical Committee of Saint-Luc University Hospital (reference nr. 2021/18FEV/086). The studies were conducted in accordance with the local legislation and institutional requirements. The participants provided their written informed consent to participate in this study.

## Author contributions

AB: Conceptualization, Formal analysis, Investigation, Methodology, Project administration, Software, Writing – original draft, Writing – review & editing. EB: Investigation, Writing – review & editing, Methodology. VJ: Investigation, Writing – review & editing. GD: Investigation, Writing – review & editing. VD: Writing – review & editing. ND: Methodology, Writing – review & editing, Software. IC: Writing – review & editing. SS: Writing – review & editing, Methodology. EM: Writing – review & editing. AS: Writing – review & editing. BM: Methodology, Writing – review & editing. LD: Methodology, Writing – review & editing, Supervision. GV: Conceptualization, Supervision, Writing – review & editing, Methodology. RET: Conceptualization, Supervision, Writing – review & editing, Methodology.

## Footnotes

1
http://stnava.github.io/ants/

2
https://github.com/hyedryn/elikopy

3
https://dipy.org/

4
https://fsl.fmrib.ox.ac.uk/fsl/fslwiki

5
https://dsi-studio.labsolver.org/
