# Supplementary material for: Locus coeruleus features are linked to vagus nerve stimulation response in drug-resistant epilepsy
Source: Front Neurosci. 2024 Feb 13;18:1296161. doi: 10.3389/fnins.2024.1296161 (PMC10926962; doi:10.3389/fnins.2024.1296161)
Supplement: Supplementary file 1 [file Data_Sheet_1.docx]

Supplementary Material

## Supplementary Figures and Tables


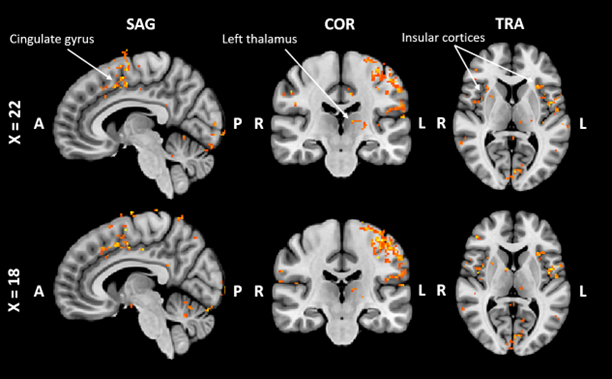


**Supplementary material 1: Sagittal, coronal, and transverse views of the statistical maps corresponding to brain response to the target stimuli during the auditory oddball task. for all patients (X = 22, top figure) and only patients with a good (>90% detection of target sounds) accuracy to the task (X = 18, bottom figure).** In order to confirm typical brain responses to the oddball task, a 2^nd^-level analysis was conducted in the Montreal Neurological Institute (MNI) space (ICBM-MNI 152, version 2009c asymmetric). Individual 1^st^-level statistical map of the target appearance was registered to the MNI space, and a random effects group analysis was conducted. Due to the small size of our sample, a lenient threshold p < 0.01 was used to report the general brain activations observed at the appearance of target sounds and ensure that the activations observed were not related to noise.

Whole-brain group-level analysis of fMRI data at a lenient statistical threshold (p<0.01, uncorrected for multiple comparisons) revealed the typical brain activations previously reported for an auditory oddball task, including the insular cortex, the anterior division of the cingulate gyrus, and the thalamus. A comprehensive table reporting the activation foci at the appearance of target sounds can be found in Supplementary material 2. A significant activation of the LC was not expected at the whole-brain group level, due to the small size of the nucleus and the limited sample size. Therefore, the LC activity was further analyzed in the space of the subject.

| Brain region  (Peak MNI coordinates) | MNI coordinates (x, y, z) | | | t-value | p-value | Cluster size |
| --- | --- | --- | --- | --- | --- | --- |
| R Supramarginal gyrus AD | 68 | -31 | 28 | 3.47965 | 0.002237 | 96 |
| R Supramarginal gyrus PD | 64 | -43 | 24 | 5.160909 | 4.10E-05 | 152 |
| R Precentral Gyrus | 54 | 5 | 15 | 5.0936 | 4.80E-05 | 328 |
| R Precentral Gyrus | 58 | 9 | 1 | 3.871333 | 0.000883 | 96 |
| R Supramarginal gyrus AD | 51 | -31 | 51 | 4.407302 | 0.000245 | 136 |
| R Middle frontal gyrus | 52 | 14 | 42 | 4.475682 | 0.000208 | 152 |
| R Temporal pole | 53 | 17 | -9 | 3.931808 | 0.000765 | 96 |
| R Insular cortex | 37 | 6 | 6 | 4.20561 | 0.000397 | 376 |
| R Middle frontal gyrus | 42 | 33 | 34 | 3.618289 | 0.001612 | 136 |
| R Middle frontal gyrus | 40 | 3 | 62 | 3.988464 | 0.000668 | 120 |
| R Superior frontal gyrus | 25 | 1 | 55 | 6.848522 | 1.00E-06 | 440 |
| R Temporal occipital fusiform | 28 | -63 | -19 | 5.576286 | 1.60E-05 | 424 |
| R Occipital fusiform gyrus | 34 | -69 | -23 | 5.658057 | 1.30E-05 | 352 |
| R Superior frontal gyrus | 27 | -1 | 63 | 4.253577 | 0.000354 | 160 |
| R Cerebellum | 20 | -56 | -20 | 5.302732 | 2.90E-05 | 680 |
| R Precentral Gyrus | 7 | -2 | 62 | 5.979005 | 6.00E-06 | 784 |
| R Superior frontal gyrus | 10 | 12 | 66 | 4.502155 | 0.000196 | 112 |
| R Lingual gyrus | 14 | -73 | -13 | 4.872447 | 8.10E-05 | 144 |
| Cingulate gyrus AD | 0 | -3 | 50 | 8.15703 | 0 | 3984 |
| R Superior frontal gyrus | 8 | 5 | 66 | 4.513535 | 0.00019 | 336 |
| R Supracalcarine cortex | -2 | -84 | 7 | 4.595208 | 0.000157 | 392 |
| L Superior frontal gyrus | 1 | 7 | 62 | 4.488734 | 0.000202 | 120 |
| Precuneous cortex | 0 | -49 | 64 | 4.14519 | 0.000459 | 104 |
| Left cingulate gyrus AD | -2 | -2 | 33 | 4.895652 | 7.70E-05 | 112 |
| R Lingual gyrus | -1 | -88 | -14 | 3.750914 | 0.001177 | 136 |
| L Intracalcarine cortex | -9 | -76 | 10 | 4.178679 | 0.000424 | 128 |
| Left thalamus | -10 | -21 | 9 | 4.382185 | 0.000261 | 112 |
| L Temporal occipital fusiform | -23 | -59 | -18 | 4.734857 | 0.000112 | 840 |
| L Precentral gyrus | -15 | -13 | 75 | 3.68581 | 0.001374 | 104 |
| L Precentral gyrus | -28 | -8 | 58 | 6.184498 | 4.00E-06 | 2016 |
| L Occipital fusiform gyrus | -16 | -79 | -22 | 5.315825 | 2.90E-05 | 224 |
| L Cerebellum | -16 | -75 | -44 | 5.046857 | 5.40E-05 | 144 |
| L Superior frontal gyrus | -17 | -9 | 70 | 4.039196 | 0.000592 | 120 |
| L Postcentral gyrus | -20 | -35 | 74 | 4.457869 | 0.000217 | 104 |
| L Postcentral gyrus | -36 | -30 | 61 | 6.205268 | 4.00E-06 | 8232 |
| L Occipital fusiform gyrus | -24 | -79 | -16 | 4.440164 | 0.000227 | 288 |
| L Superior frontal gyrus | -25 | -5 | 67 | 4.223297 | 0.000381 | 96 |
| L Middle frontal gyrus | -31 | 3 | 67 | 4.435038 | 0.00023 | 136 |
| L Cerebellum | -26 | -85 | -34 | 4.389474 | 0.000256 | 96 |
| L Frontal operculum cortex | -37 | 21 | 6 | 4.839891 | 8.80E-05 | 224 |
| L Cerebellum | -30 | -55 | -32 | 5.722091 | 1.10E-05 | 128 |
| L Occipital fusiform gyrus | -33 | -79 | -20 | 4.873293 | 8.10E-05 | 120 |
| L Precentral gyrus | -36 | -7 | 69 | 4.713655 | 0.000118 | 280 |
| L Middle frontal gyrus | -34 | 37 | 32 | 5.644079 | 1.30E-05 | 136 |
| L Middle frontal gyrus | -37 | 35 | 24 | 4.634315 | 0.000143 | 96 |
| L Superior parietal lobule | -38 | -48 | 65 | 4.705645 | 0.00012 | 136 |
| L Frontal operculum cortex | -47 | 10 | -4 | 4.325704 | 0.000298 | 240 |
| L Frontal orbital cortex | -37 | 25 | -5 | 4.449174 | 0.000222 | 160 |
| L Cerebellum | -37 | -63 | -25 | 4.053257 | 0.000572 | 136 |
| L Occipital fusiform gyrus | -42 | -69 | -23 | 4.3321 | 0.000294 | 144 |
| L Central opercular cortex | -49 | 0 | 6 | 6.906199 | 1.00E-06 | 216 |
| L Parietal operculum cortex | -59 | -27 | 15 | 4.538655 | 0.000179 | 888 |
| L Cerebellum | -48 | -67 | -26 | 4.597201 | 0.000156 | 216 |
| L Supramarginal gyrus, PD | -49 | -42 | 25 | 4.884823 | 7.90E-05 | 112 |
| L Inferior frontal gyrus, pars opercularis | -52 | 19 | -5 | 5.02606 | 5.60E-05 | 232 |
| L Angular gyrus | -52 | -60 | 12 | 6.049971 | 5.00E-06 | 160 |
| L Precentral gyrus | -51 | 6 | 11 | 4.332529 | 0.000293 | 128 |
| L Temporal pole | -56 | 8 | -1 | 5.201084 | 3.70E-05 | 96 |
| L Supramarginal gyrus, PD | -61 | -43 | 27 | 6.369423 | 3.00E-06 | 304 |
| L Central opercular cortex | -60 | -7 | 9 | 4.496726 | 0.000198 | 160 |
| L Planum temporale | -59 | -25 | 10 | 4.707882 | 0.00012 | 104 |

**Supplementary material 2: Brain regions activated at the appearance of target sounds (second-level analysis, all patients included) -** Activation foci for p<0.01. R: Right, L: Left, AD: Anterior Division, PD: Posterior Division.

| **(A) LC activity**  **~** | | **Response** | **Age** | **Sex** | **ASM** | **Benzo** | **Epilepsy duration** |
| --- | --- | --- | --- | --- | --- | --- | --- |
| **LEFT** | Rostral | p = 0.42  t = -2.63 | p = 0.69  t = -1.43 | p = 0.66  t = 0.79 | p = 0.73  t = -0.88 | p = 0.14  t = 0.51 | p = 0.71  t = 1.01 |
| Medial | | **p = 0.01***  t = -2.63 | p = 0.17  t = -1.43 | p = 0.44  t = 0.79 | p = 0.39  t = -0.88 | p = 0.62  t = 0.51 | p = 0.33  t = 1.01 |
| Caudal | | p = 0.59  t = 0.55 | p = 0.27  t = -0.14 | p = 0.70  t = 0.38 | p = 0.19  t = 1.38 | p = 0.41  t = -0.84 | p = 0.87  t = 0.16 |
|  | |  |  |  |  |  |  |
| **RIGHT** | Rostral | p = 0.52  t = 0.66 | p = 0.70  t = 0.39 | p = 0.65  t = -0.46 | p = 0.10  t = 1.74 | p = 0.41  t = -0.85 | p = 0.45  t = 0.78 |
| Medial | | p = 0.54  t = -0.62 | p = 0.50  t = -0.69 | p = 0.56  t = -0.60 | p = 0.67  t = 0.44 | p = 0.22  t = -1.29 | p = 0.44  t = 0.78 |
| Caudal | | p = 0.40  t = 0.87 | p = 0.22  t = -1.28 | p = 0.24  t = -1.21 | p = 0.96  t = 0.05 | p = 0.44  t = -0.79 | p = 0.06  t = 2.00 |
|  | |  |  |  |  |  |  |

| **(B) LC activity**  ~ | | **Therapy**  **Duration** | **Age** | **Sex** | **ASM** | **Benzo** | **Epilepsy duration** |
| --- | --- | --- | --- | --- | --- | --- | --- |
| **LEFT** | Rostral | p = 0.07  t = -1.95 | p = 0.23  t = 1.24 | p = 0.45  t = -0.77 | p = 0.84  t = -0.21 | p = 0.24  t = 1.21 | p = 0.65  t = -0.46 |
| Medial | | p = 0.72  t = -0.36 | p = 0.27  t = -1.13 | p = 0.48  t = 0.73 | p = 0.78  t = -0.28 | p = 0.60  t = 0.53 | p = 0.56  t = 1.30 |
| Caudal | | p = 0.78  t = 0.28 | p = 0.28  t = -1.11 | p = 0.70  t = 0.38 | p = 0.23  t = 1.23 | p = 0.43  t = -0.81 | p = 0.82  t = 0.23 |
|  | |  |  |  |  |  |  |
| **RIGHT** | Rostral | p = 0.20  t = 1.35 | p = 0.93  t = -0.09 | p = 0.73  t = -0.35 | p = 0.16  t = 1.46 | p = 0.52  t = -0.66 | p = 0.34  t = 0.98 |
| Medial | | p = 0.54  t = -0.62 | p = 0.66  t = -0.45 | p = 0.53  t = -0.64 | p = 0.52  t = 0.65 | p = 0.20  t = -1.34 | p = 0.50  t = 0.68 |
| Caudal | | p = 0.54  t = -0.61 | p = 0.66  t = -0.45 | p = 0.53  t = -0.64 | p = 0.52  t = 0.66 | p = 0.20  t = -1.34 | p = 0.50  t = 0.68 |
|  | |  |  |  |  |  |  |

| **(C) LC contrast** ~ | | **Response** | **Age** | **Sex** | **ASM** | **Benzo** | **Epilepsy duration** |
| --- | --- | --- | --- | --- | --- | --- | --- |
| **LEFT** | Rostral | p = 0.84  t = 0.20 | p = 0.58  t = 0.56 | p = 0.55  t = -0.60 | p = 0.91  t = 0.11 | p = 0.26  t = 1.16 | p = 0.60  t = -0.54 |
| Medial | | p = 0.53  t = -0.64 | p = 0.23  t = -1.24 | p = 0.63  t = -0.49 | p = 0.53  t = -0.64 | p = 0.42  t = 0.83 | p = 0.13  t = 1.57 |
| Caudal | | p = 0.19  t = 1.37 | p = 0.85  t = 0.19 | p = 0.77  t = -0.30 | p = 0.69  t = -0.41 | p = 0.17  t = 1.45 | p = 0.87  t = 0.16 |
|  | |  |  |  |  |  |  |
| **RIGHT** | Rostral | p = 0.21  t = 1.29 | p = 0.29  t = 1.10 | p = 0.43  t = -0.82 | p = 0.79  t = 0.28 | p = 0.54  t = 0.62 | p = 0.17  t = -1.44 |
| Medial | | p = 0.65  t = 0.46 | p = 0.68  t = -0.42 | p = 0.53  t = -0.65 | p = 0.81  t = -0.24 | p = 0.38  t = 0.89 | p = 0.62  t = 0.51 |
| Caudal | | **p = 0.03 ***  t = 2.39 | p = 0.61  t = 0.53 | p = 0.79  t = -0.27 | p = 0.79  t = 0.27 | p = 0.27  t = 1.13 | p = 0.64  t = -0.48 |

| **(D) LC contrast ~** | | **Therapy**  **Duration** | **Age** | **Sex** | **ASMs** | **Benzo** | **Epilepsy duration** |
| --- | --- | --- | --- | --- | --- | --- | --- |
| **LEFT** | *Rostral* | p = 0.81  t = 0.25 | p = 0.64  t = 0.47 | p = 0.53  t = -0.63 | p = 0.99  t = 0.007 | p = 0.26  t = 1.18 | p = 0.62  t = -0.49 |
| Medial | | **p = 0.03***  t = 2.28 | p = 0.08  t = -1.87 | p = 0.83  t = -0.21 | p = 0.32  t = -1.03 | p = 0.16  t = 1.47 | p = 0.09  t = 1.78 |
| Caudal | | p = 0.29  t = 1.09 | p = 0.86  t = -0.17 | p = 0.63  t = -0.49 | p = 0.33  t = -1.00 | p = 0.14  t = 1.55 | p = 0.66  t = 0.44 |
|  | |  |  |  |  |  | O |
| **RIGHT** | Rostral | p = 0.92  t = -0.09 | p = 0.35  t = 0.96 | p = 0.31  t = -1.07 | p = 0.96  t = -0.04 | p = 0.63  t = 0.48 | p = 0.26  t = -1.18 |
| Medial | | **p = 0.04***  t = 2.26 | p = 0.31  t = -1.05 | p = 0.51  t = -0.67 | p = 0.38  t = -0.90 | p = 0.16  t = 1.46 | p = 0.45  t = 0.77 |
| Caudal | | p = 0.38  t = 0.89 | p = 0.93  t = 0.09 | p = 0.54  t = -0.62 | p = 0.61  t = -0.51 | p = 0.32  t = 1.03 | p = 0.99  t = -0.01 |

| **(E) Diffusion metric ~** | | **Response** | **Age** | **Sex** | **ASMs** | **Benzo** | **Epilepsy duration** | |
| --- | --- | --- | --- | --- | --- | --- | --- | --- |
| **LEFT** | *wFVF* | **p = 0.02***  t = 2.61 | **p = 9.18e-5***  t = 5.98 | **p = 0.002***  t = -4.11 | p = 0.48  t = 0.72 | **p = 0.04***  t = -2.37 | p = 0.05  t = -2.24 |  |
| FA | | p = 0.83  t = 0.22 | **p =** **0.01***  t = 3.04 | p = 0.11  t = -1.75 | p = 0.56  t = 0.59 | p = 0.31  t = -1.06 | p = 0.09  t = -1.85 |  |
| MD | | **p = 0.001***  t = -4.31 | **p = 0.006***  t = -3.38 | p = 0.82  t = -0.22 | p = 0.07  t = -1.98 | p = 0.06  t = 2.06 | p = 0.07  t = 1.99 |  |
| AD | | **p = 0.02***  t = -2.64 | p = 0.54  t = -0.62 | p = 0.29  t = -1.11 | p = 0.29  t = 0.89 | p = 0.39  t = 0.89 | p = 0.62  t = 0.51 |  |
| RD | | **p = 1.49e-5***  t = -7.33 | **p = 1.67e-6***  t = -9.21 | p = 0.07  t = 1.97 | **p =** **0.004***  t = -3.57 | **p = 0.001***  t = 4.40 | **p = 3e-4***  t = 5.07 |  |
|  | |  |  |  |  |  |  |  |
| **RIGHT** | *wFVF* | p = 0.10  t = 1.82 | p = 0.06  t = 2.15 | p = 0.97  t = 0.03 | p = 0.44  t = 0.80 | p = 0.59  t = -0.56 | p = 0.31  t = -1.07 |  |
| FA | | p = 0.79  t = 0.26 | p = 0.3  t = 1.09 | p = 0.96  t = 0.05 | p = 0.85  t = 0.19 | p = 0.67  t = -0.43 | p = 0.28  t = -1.14 |  |
| MD | | **p = 0.01***  t = -2.95 | p = 0.2  t = -1.37 | p = 0.29  t = -1.12 | p = 0.13  t = -1.65 | p = 0.93  t = 0.08 | p = 0.65  t = 0.47 |  |
| AD | | p = 0.07  t = -1.98 | p = 0.65  t = -0.47 | p = 0.38  t = -0.91 | p = 0.24  t = -1.25 | p = 0.91  t = 0.12 | p = 0.85  t = -0.19 |  |
| RD | | **p = 0.003***  t = -3.74 | **p = 0.036***  t = -2.42 | p = 0.26  t = -1.20 | p = 0.09  t = -1.87 | p = 0.99  t = 0.003 | p = 0.21  t = 1.35 |  |
|  | |  |  |  |  |  |  |  |

| **(F) Diffusion metric ~** | | **Therapy duration** | **Age** | **Sex** | **ASMs** | **Benzo** | **Epilepsy duration** |
| --- | --- | --- | --- | --- | --- | --- | --- |
| **LEFT** | wFVF | p = 0.17  t = -1.48 | p = 4.3e-4  t = 4.93 | p = 8.1e-3  t = -3.22 | p = 0.74  t = 0.34 | p = 0.06  t = -2.1 | p = 0.10  t = -1.81 |
| FA | | p = 0.70  t = -0.39 | p = 0.01  t = 3.07 | p = 0.11  t = -1.74 | p = 0.57  t = 0.59 | p = 0.30  t = -1.09 | p = 0.09  t = -1.86 |
| MD | | p = 0.32  t = 1.03 | p = 0.09  t = -1.88 | p = 0.59  t = -0.55 | p = 0.39  t = -0.89 | p = 0.21  t = 1.33 | p = 0.29  t = 1.11 |
| AD | | p = 0.51  t = 0.67 | p = 0.79  t = -0.27 | p = 0.25  t = -1.21 | p = 0.56  t = -0.60 | p = 0.49  t = 0.71 | p = 0.78  t = 0.29 |
|  | |  |  |  |  |  |  |
| **RIGHT** | wFVF | p = 0.16  t = -1.51 | p = 0.08  t = 1.95 | p = 0.93  t = 0.09 | p = 0.73  t = 0.35 | p = 0.59  t = -0.55 | p = 0.31  t = -1.08 |
| FA | | p = 0.27  t = -1.17 | p = 0.26  t = -1.17 | p = 0.94  t = 0.08 | p = 0.85  t = 0.19 | p = 0.59  t = -0.55 | p = 0.24  t = -1.25 |
| MD | | p = 0.25  t = 1.22 | p = 0.41  t = -0.86 | p = 0.36  t = -0.95 | p = 0.49  t = -0.71 | p = 0.99  t = 8e-3 | p = 0.69  t = 0.40 |
| AD | | p = 0.65  t = 0.53 | p = 0.83  t = -0.22 | p = 0.42  t = -0.83 | p = 0.54  t = 0.62 | p = 0.98  t = 0.02 | p = 0.89  t = -0.14 |

# Supplementary material 3: Linear models (LM) : (A) effect of therapy duration on LC activity (all patients included), and (B) effect of therapy duration on the diffusion metrics within the LC-hippocampus connections, after controlling for age, sex, ASM intake, benzodiazepines intake and epilepsy duration.


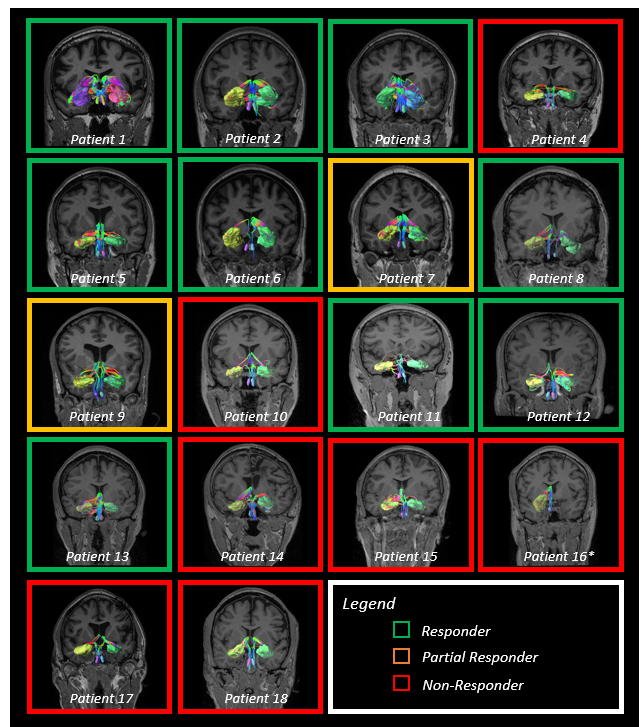


**Supplementary material 4: Tractography of left and right LC-hippocampus connections in all patients -** Tractography of subject 16 was only conducted for the left LC-hippocampus connections, due to an amygdalohippocampectomy performed in the right hemisphere.
